# Platelet function is disturbed by the angiogenesis inhibitors sunitinib and sorafenib, but unaffected by bevacizumab

**DOI:** 10.1007/s10456-018-9598-5

**Published:** 2018-03-12

**Authors:** Maudy Walraven, Marjolein Y. V. Homs, Astrid A. M. van der Veldt, Henk Dekker, Jose Koldenhof, Richard Honeywell, Arjan Barendrecht, Silvie A. E. Sebastian, Naomi Parr, Arnold C. Koekman, Emile E. Voest, Mark Roest, Suzanne J. A. Korporaal, Henk M. W. Verheul

**Affiliations:** 1 Department of Medical Oncology, Cancer Center Amsterdam, VU University Medical Center, De Boelelaan, 1117, 1081 HV Amsterdam, The Netherlands; 2 Department of Medical Oncology, University Medical Center Utrecht, Utrecht University, Utrecht, The Netherlands; 3 Department of Medical Oncology, Erasmus MC Cancer Institute, Rotterdam, The Netherlands; 4 Department of Clinical Chemistry and Haematology, University Medical Center Utrecht, Utrecht University, Utrecht, The Netherlands; 5 Department of Medical Oncology, The Netherlands Cancer Institute, Amsterdam, The Netherlands

**Keywords:** Platelet function, TKIs, VEGF, Bleeding

## Abstract

**Introduction:**

At the clinical introduction of antiangiogenic agents as anticancer agents, no major toxicities were expected as merely just endothelial cells (ECs) in tumors would be affected. However, several (serious) toxicities became apparent, of which underlying mechanisms are largely unknown. We investigated to what extent sunitinib (multitargeted antiangiogenic tyrosine kinase inhibitor (TKI)), sorafenib (TKI) and bevacizumab [specific antibody against vascular endothelial growth factor (VEGF)] may impair platelet function, which might explain treatment-related bleedings.

**Materials and methods:**

In vitro, the influence of sunitinib, sorafenib, and bevacizumab on platelet aggregation, P-selectin expression and fibrinogen binding, platelet–EC interaction, and tyrosine phosphorylation of c-Src was studied by optical aggregation, flow cytometry, real-time perfusion, and western blotting. Ex vivo, platelet aggregation was analyzed in 25 patients upon sunitinib or bevacizumab treatment. Concentrations of sunitinib, VEGF, and platelet and EC activation markers were measured by LC–MS/MS and ELISA.

**Results:**

In vitro, sunitinib and sorafenib significantly inhibited platelet aggregation (20 μM sunitinib: 71.3%, *p* < 0.001; 25 μM sorafenib: 55.8%, *p* = 0.042). Sorafenib and sunitinib significantly inhibited P-selectin expression on platelets. Exposure to both TKIs resulted in a reduced tyrosine phosphorylation of c-Src. Ex vivo, within 24 h sunitinib impaired platelet aggregation (83.0%, *p* = 0.001, *N* = 8). Plasma concentrations of sunitinib, VEGF, and platelet/EC activation markers were not correlated with disturbed aggregation. In contrast, bevacizumab only significantly impaired platelet aggregation in vitro at high concentrations, but not ex vivo.

**Conclusion:**

Sunitinib significantly inhibits platelet aggregation in patients already after 24 h of first administration, whereas bevacizumab had no effect on aggregation. These findings may explain the clinically observed bleedings during treatment with antiangiogenic TKIs.

**Electronic supplementary material:**

The online version of this article (10.1007/s10456-018-9598-5) contains supplementary material, which is available to authorized users.

## Introduction

Interference with vascular endothelial growth factor (VEGF) signaling and subsequent tumor neovascularization by the use of targeted agents has shown beneficial effects for patients with various tumor types [1–5]. Specific antibody-based VEGF inhibitors, like bevacizumab, have been developed, as well as small-molecule antiangiogenic multityrosine kinase inhibitors (TKIs) interfering with VEGF signaling, like sunitinib and sorafenib. It was not expected that these antiangiogenic agents would cause severe toxicities, as merely non-quiescent endothelial cells (ECs) present in the tumor would be disturbed [6]. However, several (major) bleeding complications, such as subungual splinter hemorrhage, epistaxis, and gastrointestinal, pulmonary, and intracerebral bleedings, have been clinically observed (all-grade bleeding events: sunitinib: 19.3%, sorafenib: 13.5%, bevacizumab: 25.0–30.4%; high-grade bleeding events: sunitinib: 3.0%, sorafenib: 2.2%, bevacizumab: 2.8–3.5%) [7–14]. Of the observed bleeding events, splinter bleedings are more frequently seen during TKI treatment.

The underlying mechanisms of these treatment-related bleeding complications have not been elucidated yet.

For adequate coagulation, coagulation factors and enzymes, ECs and platelets (thrombocytes) are important [15]. Platelets play a specific role in clot formation, because they immediately adhere to the damaged endothelial layer of the vessel wall upon detection of a breach, where they become activated, aggregate and release their content [15, 16]. Relatively small differences in this orchestrated interplay may cause significant disturbances leading to bleeding complications.

It has become apparent that platelets not only play a role in arrest of bleeding but are also involved in vascular integrity and vessel repair [17, 18]. Since VEGF is crucial for maintaining the integrity of the microvasculature, it has been postulated that blockade of the VEGF pathway leads to compromised capacity of ECs for cell repair [17, 19, 20], which has been proposed as a mechanism for the observed vascular toxicity. Previously, we found that VEGF is transported by platelets and released upon platelet activation [21]. In addition, it became apparent that bevacizumab is taken up by platelets and neutralizes circulating platelet-VEGF [22]. Based on these findings and the clinically observed bleeding complications, we hypothesized that antiangiogenic treatment may disturb platelet function which may be the cause behind the bleeding complications. To study this hypothesis, we evaluated the influence of the antiangiogenic agents sunitinib, sorafenib, and bevacizumab on platelet function both in vitro and ex vivo using platelets from patients with renal cell carcinoma (RCC) and non-small cell lung carcinoma (NSCLC).

## Materials and methods

Detailed information is described in supplementary Materials and methods.

### Healthy volunteers

Informed consent was obtained before blood collection. Healthy volunteers did not take any medication in the prior 10 days. In vitro experiments were performed to study the effect of sunibitinib (10 or 20 μM), sorafenib (5, 10, 25 μM), and bevacizumab (50, 100 or 250 μg/ml) on platelet aggregation. Clinically achieved concentrations for sunitinib (50 mg/day) are approximately 65 ng/ml (0.16 μM) [23], for sorafenib (400 mg bid) around 5 mg/l (10.7 μM) [24, 25], and for bevacizumab (10 mg/kg) up to 300 μg/ml [26]. We chose these high concentrations with regard to the relative short incubation time. Furthermore, the influence of the angiogenesis inhibitors was examined on platelet–EC adhesion (measured by real-time perfusion) and on surface P-selectin expression and fibrinogen binding to αIIbβ3 (measured by flow cytometry). Finally, tyrosine phosphorylation of Src was studied in the presence of both TKIs sunitinib and sorafenib (measured by western blotting).

Additional information regarding used reagents and antibodies and the methods used is described in supplementary materials and methods.

### Patients

Blood was drawn by venipuncture from patients with RCC before and during treatment with sunitinib and in patients with NSCLC before and after a single administration of bevacizumab. The studies were approved by the institutional review boards. Patients were required to sign informed consent prior to participation.

Time points for collection of blood in patients treated with sunitinib were: pretreatment, 24 h, 3 weeks, and 6 weeks after start of treatment. Platelet aggregation experiments and measurements of the concentrations of sunitinib [measured by liquid chromatography–tandem mass spectrometry (LC–MS/MS)], of VEGF, and of the activation markers of platelets and ECs [measured by enzyme-linked immunosorbent assay (ELISA)] were performed at the different time points. Treatment-related toxicity was reported during the first 6 weeks of treatment with sunitinib. Common Terminology Criteria for Adverse Events (CTCAE) were used to grade bleeding events.

Treatment with chemotherapy or biologicals was not allowed in the previous 28 days before start sunitinib.

Time points for collection of blood samples in patients treated with bevacizumab were: before, and 5 h and 3 to 5 days after the administration of bevacizumab. Prior to their scheduled therapy, these patients received a single dose of 15 mg/kg bevacizumab as part of an imaging study [27]. In this study, a week prior to infusion patients underwent a dynamic PET-CT study with both [15O]H2O and [11C]docetaxel. Platelet aggregation was performed at the different time points.

Additional information regarding the methods used is described in supplementary methods.

### Statistical analysis

For statistical analysis, SPSS (version 21.0; SPSS INC., Chigaco, IL, USA) was used. Paired *t* tests were performed to compare the absolute values of aggregation levels, the concentration of platelet and EC activation markers, the VEGF concentrations and the platelet counts over time, and the expression of P-selectin on the platelet membrane and the fibrinogen binding to GPIIb/IIIa. Independent-sample *t* tests were performed to compare impaired platelet aggregation in patients with and without bleeding events. With the Pearson correlation coefficient, the concentrations of sunitinib in plasma and serum and the changes in platelet counts were correlated with impaired platelet aggregation. In addition, the concentrations of platelet activation markers were correlated with platelet counts. Defined significance level is *p* < 0.05. Reported *p* values are two-sided.

## Results

### In vitro

#### Inhibition of platelet aggregation upon in vitro exposure to sunitinib, sorafenib, and bevacizumab

**Fig. 1:**
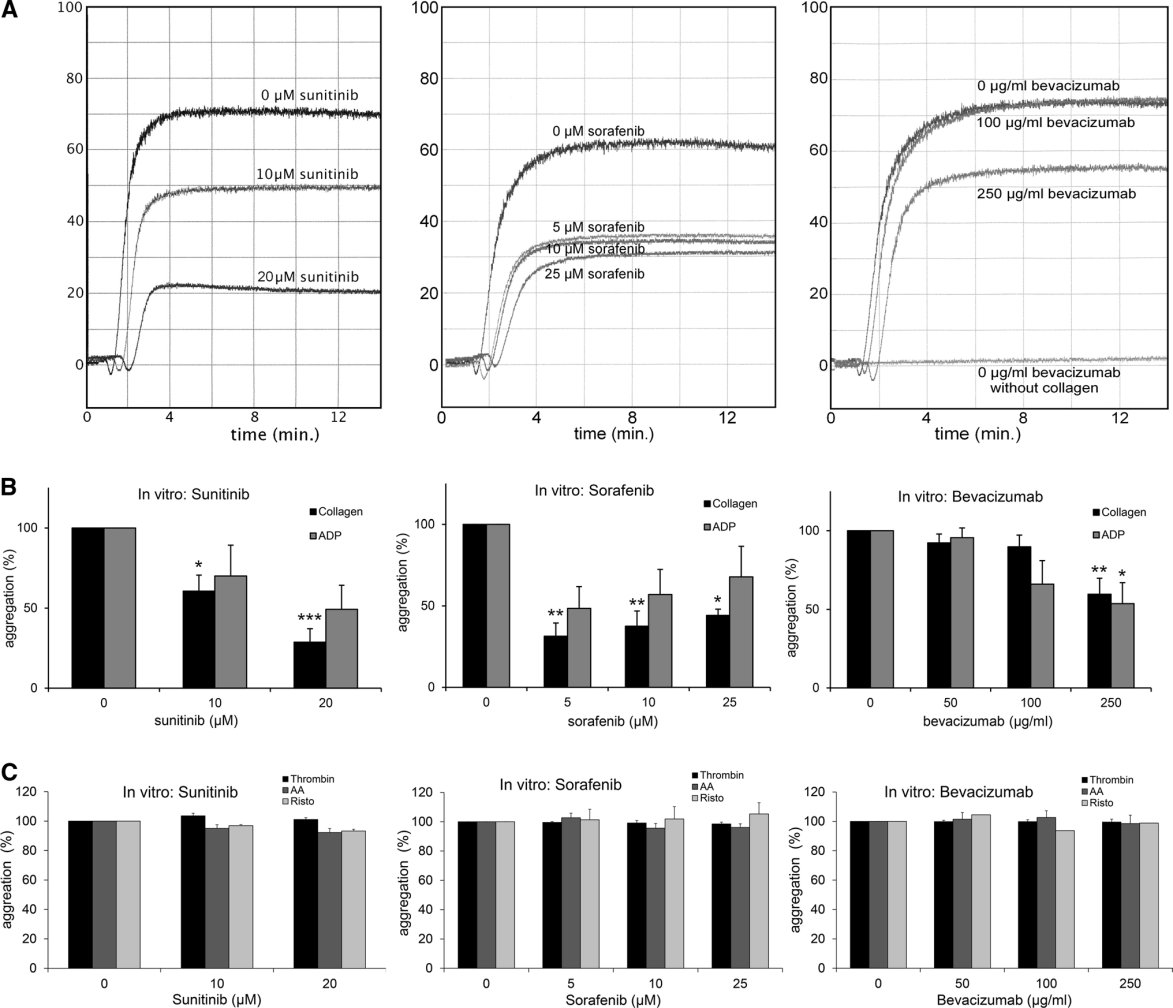
Disturbance of platelet aggregation due to antiangiogenic treatment with sunitinib, sorafenib and bevacizumab in vitro. **a** Representative aggregation curves of in vitro platelet aggregation experiments with collagen after incubation of platelets with sunitinib, sorafenib or bevacizumab.(Left graph: 0, 10, 20 μM sunitinib, middle graph: 0, 5, 10, 25 μM sorafenib, right graph: 0, 100, 250 μg/ml bevacizumab). On the *X*-axis time is represented in minutes. On the *Y*-axis the percentage of aggregation is represented. **b** In vitro platelet aggregation after incubation with sunitinib, sorafenib or bevacizumab. Platelets were activated with collagen (0.25–1.0 μg/ml) or ADP (2.5–10 μM) (*N* = 3–6). On the *X*-axis the concentration of the angiogenesis inhibitor is represented, on the *Y*-axis the percentage of aggregation compared to control. **c** In vitro platelet aggregation induced by thrombin (0.125–0.25 U/ml), arachidonic acid (0.25 mM) or ristocetin/vWF (25 mg/ml/5 μ/ml) after incubation with sunitinib, sorafenib or bevacizumab (*N* = 3–5). On the *X*-axis the concentration of the angiogenesis inhibitor is represented, on the *Y*-axis the percentage of aggregation compared to control. The error bars represent the standard error of mean. * = *p* ≤ 0.05, ** = *p* ≤ 0.01, *** = *p* ≤ 0.001

Platelet aggregation was impaired in vitro when platelets of healthy volunteers were pre-incubated with sunitinib, sorafenib, and bevacizumab and activated by collagen or ADP (Fig. 1a, b). Collagen-induced platelet aggregation was decreased with 39.4% (range 28.6–59.3, *p* = 0.019) and 71.3% (range 58.2–87.0, *p* < 0.001) by 10 and 20 μM sunitinib, respectively. Sorafenib significantly impaired platelet aggregation at a dose of 5, 10, and 25 μM with 68.6% (range 41.0–84.6, *p* = 0.002), 62.4% (range 42.6–85.7, *p* = 0.005), and 55.8% (range 49.1–62.3, *p* = 0.042), respectively. Bevacizumab impaired the collagen activating pathway of platelets, dose dependently, by 40.3% (range 19.2–70.6, *p* = 0.003) at the highest used concentration of 250 μg/ml, but no significant inhibition at lower concentrations of bevacizumab was detected (Fig. 1b).

ADP-induced platelet aggregation was inhibited by 50.8% when exposed to 20 μM sunitinib (range 30.9–80.2, *p* = 0.098), by 51.5% (range 23.3–90.8, *p* = 0.067) when exposed to 5 μM sorafenib and by 46.4% (range 0–78.3, *p* = 0.042) when exposed to 250 μg/ml bevacizumab, respectively (Fig. 1b).

No significant inhibition of aggregation or agglutination was observed with the agonists thrombin, arachidonic acid, or ristocetin/vWF (Fig. 1c).

#### Impact of sunitinib and sorafenib on agonist-induced P-selectin expression and fibrinogen binding

**Fig. 2:**
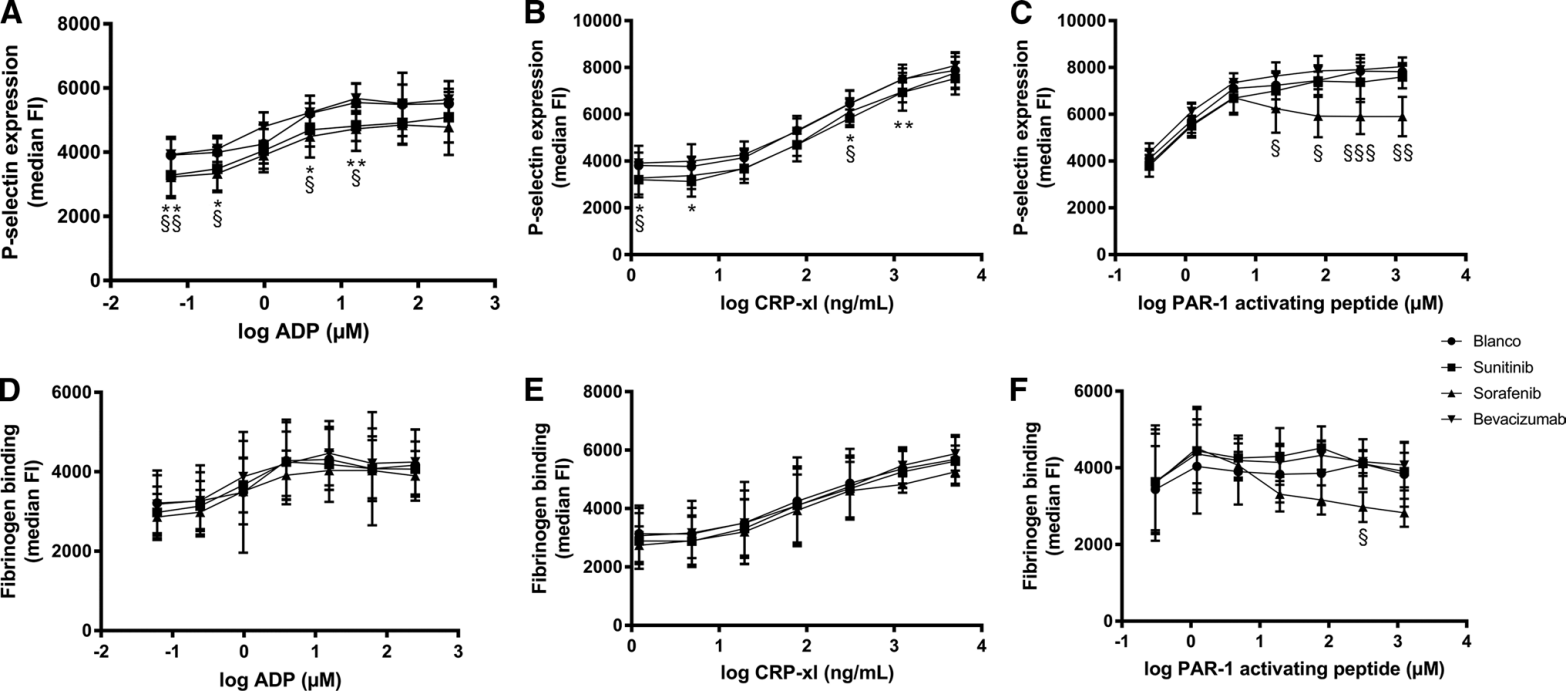
In vitro expression of P-selectin and binding of fibrinogen on platelets after platelet activation by ADP, CRP-xL or PAR-1 activating peptide in the presence of sunitinib, sorafenib and bevacizumab. **a** Expression of P-selectin after activation of platelets with ADP. **b** Expression of P-selectin after activation of platelets with CRP-xL. **c** Expression of P-selectin after activation of platelets with PAR-1 AP. **d** Fibrinogen binding after activation of platelets with ADP. **e** Fibrinogen binding after activation of platelets with CRP-xL. **f** Fibrinogen binding after activation of platelets with PAR-1 AP. On the *X*-axis are the log scales of the concentrations of the agonists used (ADP: µM, CRP-xL: ng/ml, PAR-1 AP: µM). On the *Y*-axis the median fluorescence intensity is expressed. *N* = 4. The error bars represent the standard deviation. Sunitinib: * = *p* ≤ 0.05, ** = *p* ≤ 0.01. Sorafenib: § = *p* ≤ 0.05, §§ = *p* ≤ 0.01, §§§ = *p* ≤ 0.001

In the presence of sunitinib, significant inhibition of P-selectin expression, as a measure of granule secretion, was observed after both ADP- and CRP-xL-induced platelet activation, but not with PAR1-AP as agonist (Fig. 2a–c). Interestingly, sunitinib also seemed to affect basal P-selectin expression of unstimulated platelets (data not shown). In the presence of sorafenib, we observed a significant inhibition of P-selectin expression after activation of platelets by ADP, CRP-xL, and PAR1-AP. Bevacizumab had no effect on P-selectin expression. Fibrinogen binding to GPIIb/IIIa was significantly disturbed due to the presence of sorafenib after activation of platelets with PAR1-AP, but not with ADP or CRP-xL (Fig. 2d–f). Bevacizumab and sunitinib had no effect on fibrinogen binding.

#### No disturbance of platelet–endothelial cell interaction after incubation with sunitinib sorafenib or bevacizumab

To investigate whether sunitinib, sorafenib, and bevacizumab interfere with the interaction between platelets and ECs, we studied this interaction by real-time perfusion. Platelet adherence to secreted vWF strings was not compromised by incubation of platelets with 5 or 10 μM sunitinib, with 10 μM sorafenib or with 100 or 250 μg/ml bevacizumab compared to control conditions (Supplemental Fig. 1). In addition, pre-incubation of ECs with 5 μM sunitinib did not alter the interaction between platelets and ECs. These data suggest that the antiangiogenic agents do not interfere with the interaction between platelets and ECs.

#### Analysis of phosphorylation of c-Src in platelets in the presence of sunitinib and sorafenib

Since sunitinib and sorafenib are multitargeted tyrosine kinase inhibitors, we studied whether these TKIs were able to inhibit tyrosine phosphorylation in platelets. Indeed, a small reduction in the phosphorylation of the tyrosine kinase c-Src was observed in the presence of sunitinib (20 µM) or sorafenib(25 µM) after activation of platelets with collagen by Western blotting (Supplemental Fig. 2).

### Ex vivo

#### Inhibition of platelet aggregation ex vivo during sunitinib and bevacizumab treatment

Patient characteristics and platelet counts are shown in Table 1. Seventeen patients with RCC and eight patients with NSCLC were included in the study prior to start of treatment with sunitinib or prior to the single administration of bevacizumab, respectively. Additional clinical information can be found in supplementary data. Platelet aggregation could not be studied with the platelet agonist collagen in 9 out of 17 RCC patients (technical problems in two patients; predefined 30% baseline aggregation level was not reached before start of treatment in seven patients (one patient had a platelet count < 1 × 10^11^ platelets/L). In 4 out of 17 patients, the baseline aggregation level was not reached with ADP (one patient had a platelet count < 1 × 10^11^ platelets/L) and in two patients technical problems disabled the analysis. In two out of eight NSCLC patients receiving bevacizumab, the 30% baseline aggregation level was not reached with collagen before start of treatment, and in one patient the baseline aggregation level was not reached with ADP (Supplemental Table 1A, B).

**Table 1:** Baseline demographics and bleeding events of patients treated with sunitinib and bevacizumab

	Sunitinib	Bevacizumab
*N*	17	8
Male/female	10/7	5/3
Mean age in years (range)	59 (42–73)	57 (47–70)
RCC	17	0
NSCLC	0	8
Mean platelet count pretreatment * 10^9^ (range)	359 (49–930)	212 (106–324)
Bleeding event	8	–
Grade one	6	–
Grade three	1	–
Grade four	1	–

Renal cell cancer (RCC), non-small cell lung carcinoma (NSCLC)

*N* is the number of patients

**Fig. 3:**
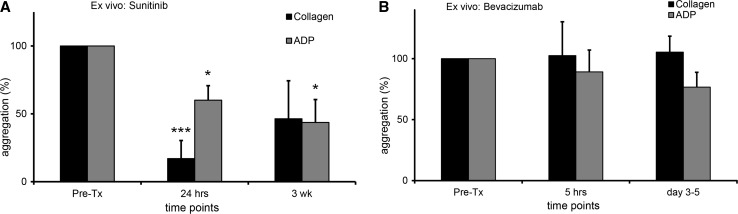
Influence on platelet aggregation of the antiangiogenic agents sunitinib and bevacizumab in patients. **a** Ex vivo platelet aggregation 24 h and 3 weeks after the first administration of sunitinib. PRP was activated with ADP (2.5–10 μM) or collagen (0.25–1.0 μg/ml). On the *X*-axis the time points are represented, on the *Y*-axis the percentage of aggregation compared to pretreatment (Collagen: pretreatment *N* = 8; 24 h *N* = 8, 3wk *N* = 5. ADP; pretreatment *N* = 11; 24 h: *N* = 11; 3wk *N* = 7)*. **b** Ex vivo platelet aggregation on day one, and 3 to 5 days after the administration of bevacizumab. PRP was activated with ADP or collagen. On the *X*-axis the time points are represented, on the *Y*-axis the percentage of aggregation compared to pretreatment (Collagen: pretreatment *N* = 6, 5 h: *N* = 4, 3–5 days: *N* = 6. ADP: pretreatment *N* = 7, 5 h: *N* = 5, 3–5 days *N* = 7). The error bars represent the standard error of mean. * = *p* ≤ 0.05, *** = *p* ≤ 0.001. *One patient with sunitinib had thrombocytosis, and we were unable to dilute the PRP beneath 6 × 10^11^ platelets/L (we therefore kept the concentration equivalent for all visits)

Twenty-four hours after start of sunitinib treatment, significantly inhibited platelet aggregation was observed for collagen stimulated platelets: 83.0% inhibition (mean, range minus 9.2–plus 100.0%, *p* = 0.001, *N* = 8 patients), and for ADP, 40.0% inhibition (mean, range minus 13.3–plus 100.0%, *p* = 0.011, *N* = 11), respectively (Fig. 3a). Three weeks after start of treatment, platelet aggregation was impaired with 53.7% (mean, range minus 52.5–plus 100.0%, *p* = 0.077, *N* = 5) and with 56.4% (mean, range minus 22.9–plus 100.0%, *p* = 0.041, *N* = 7) in case of stimulation with collagen and ADP, respectively (Fig. 3a). This inhibition was still observed after the two regular stop-weeks of sunitinib treatment (data not shown). Change in platelet counts was not significantly correlated with impaired platelet aggregation at 3 weeks after start of treatment (correlation coefficient with collagen: − 0.452, *p* = 0.445; correlation coefficient with ADP: − 0.419, *p* = 0.349).

Five hours and 3–5 days after the administered dose of bevacizumab, no significant inhibition of platelet aggregation was detected upon stimulation with collagen: at 5 h minus 2.5% inhibition (mean, range minus 41.3–plus 78.6%, *p* = 0.964, *N* = 4) and at 3 to 5 days minus 5.4% inhibition (mean, range minus 52.2–plus 39.3% *p* = 0.835, *N* = 6) (Fig. 3b). For ADP stimulation, at 5 h after administration of bevacizumab and at 3 to 5 days after the administration of bevacizumab, 10.8% inhibition (mean, range minus 49.0–plus 59.2%, *p* = 0.473, *N* = 5) and 23.3% inhibition (mean, range minus 8–plus 82.2, *p* = 0.072, *N* = 7) were observed (Fig. 3b).

#### Bleeding events

Within the first 6 weeks after start of sunitinib treatment, 6 out of 17 patients experienced a bleeding event. One patient had both epistaxis (grade three) and hematuria (grade one). This patient used a low molecular weight heparin (LMWH) among the co-medication. Another patient with intracranial metastases developed intracranial hemorrhage (grade four). All other events were grade-one bleedings: epistaxis (*N* = 2), both epistaxis and splinter bleeding (*N* = 1), hematoma at the injection site of LMWH (*N* = 1). No relation between these bleeding events and impaired platelet aggregation was found at 3 weeks after start of treatment. For bevacizumab, this analysis was not performed since platelet aggregation was not significantly reduced.

#### Plasma and serum concentrations of sunitinib in relation to platelet aggregation

The plasma and serum concentrations of sunitinib are shown in Supplemental Table 2. Twenty-four hours and 3 weeks after start of treatment, the plasma concentrations were 78.3 (range 20.1–146.6) and 151.8 nM (range 36.9–248.7.0), and the serum concentrations 99.2 (range 29.9–205.3) and 191.0 nM (range 35.6–291.1), respectively, which is comparable to prior reports [28]. Sunitinib was still partially detectable after two regular stop-weeks (plasma: 25.3 nM, serum: 51.4 nM). The plasma and serum concentrations were not significantly correlated with the extent of impaired platelet aggregation.

**Table 2:** Concentrations of VEGF and activation markers of platelets and endothelial cells measured in plasma of patients before, 24 h and 3 weeks after start of treatment with sunitinib

	Pre-Tx	24 h	3 week
VEGF in PPP (pg/ml)	101	110	218
*N*	17	17	9
SEM	18	23	72
vWF (μg/ml)	16.4	15.6	15.1
*N*	15	15	8
SEM	2.1	2.1	1.7
P-selectin (ng/ml)	120.1	154.3	92.4
*N*	15	15	8
SEM	13.2	32.5	10.4
Beta-TG (ng/ml)	39.1	36.8	26.9
*N*	15	15	8
SEM	5.2	7.4	6.3
RANTES (ng/ml)	4.3	4.1	3.2
*N*	15	15	8
SEM	0.6	0.8	0.7

Vascular endothelial growth factor (VEGF), platelet poor plasma (PPP), von Willebrand factor (vWF), beta-thromboglobulin (beta-TG)

*N* is the number of patients, SEM is the standard error of mean

#### Activation markers of platelets and endothelial cells during sunitinib treatment as measured by ELISA

Plasma concentrations of activation markers of platelets (beta-TG, RANTES, P-selectin) and ECs (vWF) in patients before and during sunitinib treatment are depicted in Table 2. The EC marker OPG was undetectable. No correlation between platelet markers and platelet counts was detected at 24 h. At 3 weeks, the concentration of beta-TG was significantly correlated with platelet count (correlation coefficient 0.986, *p* = 0.014). The concentration of platelet and EC markers did not significantly change during the course of treatment.

#### VEGF concentrations during treatment with sunitinib associated with platelet aggregation

VEGF concentrations in PPP before and during sunitinib treatment are shown in Table 2. Three weeks after start of treatment, the mean VEGF concentration was significantly increased compared to pretreatment (218 pg/ml (range 63–752) versus 101 pg/ml (range 23–262), *p* = 0.03). During the two regular stop-weeks, the concentration returned to the pretreatment value (79 pg/ml (range 28–214)). No significant changes in VEGF concentration were observed in serum (pretreatment: 960 pg/ml (range 94–1894), 24 h: 929 pg/ml (range 179–1787), 3 weeks: 956 pg/ml (range 464–1611), and 6 weeks: 668 pg/ml (range 279–862)), respectively. VEGF levels were also measured in isolated platelets of these patients. Pretreatment, 24 h, 3 weeks, and 6 weeks after start of treatment, VEGF was 139 pg per mg total protein (pg/mg TP) (range 26–475), 221 pg/mg TP (range 20–609), 133 pg/mg TP (range 11–242), and 149 pg/mg TP (range 33–315), respectively. Already at 24 h, an increased VEGF level was detected compared to the pretreatment level (*p* = 0.005). VEGF concentrations were not correlated with the extent of impaired platelet aggregation.

## Discussion

In this study, we investigated the hypothesis that targeted agents inhibiting VEGF signaling may disturb the function of platelets, thereby contributing to the observed treatment-related bleeding complications. We found in vitro that platelet aggregation, induced by collagen or ADP, is reduced by the TKIs sunitinib and sorafenib. Sunitinib impaired platelet function in patients as well, most likely due to a direct effect on platelets as no correlation was found with decreased platelet count (a known side-effect) [23]. In contrast, no effect of bevacizumab (monoclonal antibody against VEGF) on platelet aggregation was detected in patients, and only high concentrations had an inhibitory effect in vitro.

Within 24 h after patients started treatment with sunitinib, platelet aggregation was impaired by almost 50%. Approximately 35% of the patients (6 out of 17) experienced a bleeding complication within the first 6 weeks of sunitinib treatment. All-grade bleeding events observed in our study were higher than the percentages reported in the phase III study in patients with RCC (12%) [3] and in the phase III trial in GIST patients (7%) [4]. Interestingly, approximately 12% (2 out of 17) patients presented with a grade ≥ 3 bleeding event, which is remarkably higher than previously reported [3]. For this increased risk of high-grade bleeding complications, several possible explanations can be suggested: the bias introduced by the limited number of participating patients, or conversely, our data reflect more the frequency among real-world patients. An additional factor may be the difference in grading of bleeding events [12].

In vitro, no effect of sunitinib, sorafenib, or bevacizumab was observed on platelet aggregation induced by thrombin, arachidonic acid, or ristocetin/vWF. This might be explained by the fact that part of the collagen signaling pathway is routed via ADP signaling. Our results of the observed effect of sunitinib on platelet aggregation induced by collagen or ADP, but not by thrombin or arachidonic acid, are compliant with recently published data by Sabrkhany et al. [29].

To examine whether the impaired platelet aggregation is caused by a defect in platelet aggregation or platelet secretion, we analyzed the amount of P-selectin expressed on the membranes of activated platelets and of fibrinogen binding to GPIIb/IIIa. Significantly impaired expression of P-selectin was observed in the presence of sorafenib and to lesser extent of sunitinib, as compared to control conditions. Fibrinogen expression was only significantly reduced by sorafenib after platelet activation by PAR1-activating peptide. Bevacizumab had no effect on P-selectin expression or fibrinogen binding. These results indicate that disturbed secretion of the platelet content contributes to the decline in platelet aggregation [30]. In the patients treated with sunitinib, plasma concentrations of activation markers of platelets (P-selectin, beta-TG, RANTES) and of ECs (vWF) within the first six treatment weeks remained similar, while in contrast to the in vitro experiment, these platelets were not activated. These results might suggest an intact interaction between platelets and activated ECs, which is further supported by the in vitro observation that incubation of platelets with sunitinib or bevacizumab did not alter platelet adherence to ECs. This observation is indicative for the complex differential biology of platelet aggregation and platelet adherence to the endothelial cell layer of the vascular wall.

Interestingly, serum concentrations of sunitinib were higher compared to plasma at 24 h and 3 weeks after start of treatment. These results indicate that intracellular accumulation of sunitinib occurs not only in cancer cells [31] but in platelets as well [29]. Due to accumulation, it may exert direct effects on platelet signaling, which might resemble data concerning the effect of other TKIs on platelet signaling [16, 32–36].

While high concentrations of bevacizumab had some inhibitory effect on platelet aggregation in vitro, no significant inhibition was observed with platelets of patients up to 5 days after administration of the highest approved dose of bevacizumab. These findings may provide further insight in antiangiogenic treatment-related toxicities, and in the interplay of platelets and ECs to maintain vascular integrity. The observation that bevacizumab did not affect platelet function is in line with a previous report [37], as a dose range of bevacizumab in vitro failed to affect platelet aggregation. However, the effect of bevacizumab on platelet aggregation still remains controversial. Meyer et al. [38] concluded that bevacizumab can induce platelet aggregation and consequently degranulation through complex formation with VEGF and activation of the platelet FcγRIIa receptor, postulating that this might explain the observed thrombotic events. One possible explanation for these contradictory results could be artificial differences and bias introduced by the different pre-analytical and laboratory methods used for the analyses, such as used type of needle, type of anticoagulant, type of centrifuge and assay method, temperature during preparation, and the center performing the analysis [39, 40]. These discrepancies underline the undoubtedly intricate and multifactorial mechanisms that can cause vascular events in patients with cancer.

Significant changes in VEGF concentrations in patients were observed during a treatment cycle of sunitinib, which is in line with a previous report [41], in which the authors speculate that this effect is secondary to increased activity of HIF-1α, leading to treatment-related increases in tumor hypoxia. We have previously reported that VEGF is almost completely neutralized in PRP of patients after a single administration of bevacizumab [27]. Now we report that bevacizumab does not significantly inhibit platelet aggregation in these patients, indicating that VEGF has a limited role in the process of platelet aggregation. This finding is supported by previous reports indicating that only very high VEGF concentrations can contribute to platelet aggregation [42]. In contrast, several TKIs targeting other pathways than the VEGF signaling pathway have been shown to exert a negative effect on platelet aggregation as well [16, 32–36]. In one of these reports, it was suggested that inhibition of Src family kinases (SFKs), which are among other signaling proteins involved in platelet activation, might play an important role by affecting immunoreceptor tyrosine-based activation motif (ITAM) signaling [34]. Sunitinib is a very potent c-Src inhibitor, with approximately 60% inhibition at a concentration of 0.5 μM [43]. This effect might be further potentiated by increased intracellular concentrations, resulting in even more profound inhibition and therefore pronounced impairment of platelet aggregation. We detected lower tyrosine phosphorylation in platelets upon TKI incubation compared to control conditions. More studies are required to elucidate whether the influence of TKIs on tyrosine phosphorylation might underlie impaired platelet aggregation resulting in bleeding events.

To conclude, treatment with sunitinib significantly inhibits platelet aggregation in patients already from the first day of treatment, while no significant inhibition was observed by bevacizumab. We found potential evidence that impaired platelet function due to antiangiogenic TKI treatment might play a role in clinically observed bleedings. This insight might contribute to the development of new targeted agents with a reduced risk of treatment-related toxicity.

## Electronic supplementary material

Below is the link to the electronic supplementary material.

Supplementary material 1 (DOCX 29 kb)

Supplemental Figure 1: Platelet adherence to secreted vWF strings on stimulated endothelial cells under shear stress (by real-time perfusion). A) Platelets and HUVECs in absence of an angiogenesis inhibitor. B) Pre-incubation of platelets for 10 min with 10 μM sunitinib. C) Pre-incubation of HUVECs for 1 h with 5 μM sunitinib. The number of platelets attached per micrometer vWF string is presented in the quantification (JPEG 2621 kb)

Supplemental Figure 2: Tyrosine phosphorylation of c-Src in the presence of sunitinib and sorafenib. Platelets were stimulated with collagen after treatment with vehicle, sunitinib or sorafenib. Tyrosine phosphorylation of c-Src was determined by SDS-PAGE after immunoprecipitation from platelet lysates (left, upper panel). An antibody against c-Src was used as a control for equal lane loading (left, lower panel). Src has a molecular weight of approximately 60KDa. The graph (right panel) shows the semiquantification of tyrosine phosphorylation of c-Src. Data are expressed as percentage of total c-Src protein. (N = 3) (JPEG 34 kb)

Supplemental Table 1A: Details of unavailability of platelet aggregation data for the agonists ADP and collagen from patients treated with sunitinib (A). 1 = Distinct thrombocytopenia; 2 = Interruption or discontinuation due to toxicity/progressive disease; 3 = Technical problems; 4 = No blood was drawn; 5 = Pretreatment aggregation level below 30%. Use of co-medication that might influence hemostasis are included (PDF 120 kb)

Supplemental Table 1B: Details of unavailability of platelet aggregation data for the agonists ADP and collagen from patients treated with bevacizumab (B). 1 = Distinct thrombocytopenia; 2 = Interruption or discontinuation due to toxicity/progressive disease; 3 = Technical problems; 4 = No blood was drawn; 5 = Pretreatment aggregation level below 30%. Use of co-medication that might influence hemostasis are included (PDF 119 kb)

Supplemental Table 2: Concentrations of sunitinib in plasma and in serum, measured by LC–MS/MS at 24 h and 3 weeks after start of treatment. N is the number of patients, SEM is the standard error of mean (PDF 142 kb)
