# Supplementary material for: Platelet function is disturbed by the angiogenesis inhibitors sunitinib and sorafenib, but unaffected by bevacizumab
Source: Angiogenesis. 2018 Mar 12;21(2):325–34. doi: 10.1007/s10456-018-9598-5 (PMC5878190; doi:10.1007/s10456-018-9598-5)
Supplement: Supplementary file 1 — Supplementary material 1 (DOCX 29 kb) [file 10456_2018_9598_MOESM1_ESM.docx]

**Platelet function is disturbed by the angiogenesis inhibitors sunitinib and sorafenib, but unaffected by bevacizumab**

Maudy Walraven, Marjolein Y.V.Homs, Astrid A.M. van der Veldt, Henk Dekker, Jose Koldenhof, Richard Honeywell, Arjan Barendrecht, Silvie A.E. Sebastian, Naomi Parr, Arnold C. Koekman, Emile E. Voest, Mark Roest, Suzanne J.A. Korporaal, Henk M.W. Verheul

Supplementary materials and methods:

**Reagents and antibodies**

Antiangiogenic agents for *in vitro* studies included sunitinib, provided by Pfizer (Capelle aan de IJssel, The Netherlands), sorafenib from JS Res Chemical Trading (Wesel, Germany) and bevacizumab which was obtained from our pharmacy.

Platelet activation: prostacyclin (PGI2) was obtained from Cayman Chemical (Ann Arbor, MI, USA), fibrillar equine collagen I (Horm collagen) from Takeda (Linz, Austria), ADP, human fibrinogen, protease inhibitor mixture, sodium vanadate (NaVO3) and bovine serum albumin fraction V from Sigma-Aldrich (Zwijndrecht, The Netherlands), crosslinked collagen related peptide (CRP-xL) from Collagen Toolkits (University of Cambridge, Cambridge, UK), thrombin from STAGO (Leiden, The Netherlands), arachidonic acid from Bio/data corporation (Horsham, PA, USA), ristocetin from American Biochemical and Pharmaceuticals Ltd (Marlton, NJ, USA), and protease activating receptor (PAR) 1 activating peptide SFFLRN (PAR1-AP) from Bachem (Weil am Rhein, Germany). Von Willebrand Factor (vWF) was a kind gift from Dr.P Lenting, INSERM U770 (Paris, France).

Flow cytometric analysis: RPE-conjugated anti-P-selectin (AK4) and APC-conjugated anti-GP1b (HIP1) antibodies were purchased from BD Biosciences (Franklin Lakes, NJ, USA), FITC-conjugated rabbit anti-human fibrinogen was obtained from DAKO (Glostrup, Denmark).

Measurement of tyrosine phosphorylation of c-Src: anti c-src (sc-8056) and anti-scr (sc-19) from Santa Cruz Biotechnology (Dallas, TX, USA),and anti-phosphotyrosine monoclonal antibody clone 4G10 from Millipore (Amsterdam, The Netherlands). Protein G-Sepharose was purchased from Amersham (Uppsala, Sweden).

Liquid chromatography-tandem mass spectrometry (LC-MS/MS): analytical solvents such as acetonitrile, formic acid and methanol were purchased from Biosolve BV (Valkenswaard, The Netherlands). HPLC grade water was obtained by a MilliQ water purification system (Millipore, The Netherlands).

Platelet-endothelial adhesion assay: EBM2 (endothelial basal medium) and EGM-2 Single-Quots were obtained from Lonza, (Verviers, Belgium), Medium 199 (Hyclone) was purchased from Invitrogen (Breda, The Netherlands).

Analysis of activation markers of ECs and platelets: Capture and detection antibodies against beta-thromboglobulin (beta-TG) (NAP-2) (MAB393, BAF393), P-selectin (DuosetDy137 mouse anti-human P-selectin and biotinylated sheep anti-human P-selectin), RANTES (CCL5) (MAB278, AB-278-NA) and osteoprotegerin (OPG) (MAB8051, BAF805) were obtained from R&D systems (Abingdon, United Kingdom) and those against von Willebrand factor (vWF) (A0082, P0226), and streptavidin-HRP (P0449) were purchased from DAKO (Glostrup, Denmark). Supersignal West pico chemiluminescent substrate was purchased from Thermo Scientific (Waltham, MA, USA). Phosphate buffered salt pH 7.4 (PBS) was produced in-house by the pharmacy department.

VEGF analysis: R&D ELISA Kit was ordered at R&D Systems (Abingdon, United Kingdom), Mammalian Protein Extraction Reagent (M-PER) lysis buffer and the Micro BCA Kit were obtained from Thermo Fischer Scientific (Rockford, IL, USA).

**Healthy volunteers: isolation of platelets**

Informed consent was obtained before blood collection. Healthy volunteers did not take any medication in the prior ten days. Freshly drawn blood was collected by free-flow into 1/10 volume of 130mM trisodium citrate. Platelet rich plasma (PRP) was prepared by centrifugation (156x *g*, 15 min, 20°C). To prevent platelet activation during further centrifugation the pH of PRP was lowered to 6.5 by addition of 0.1 volume ACD (2.5% trisodium citrate, 1.5% citric acid, 2% D-glucose). A platelet pellet was obtained by centrifugation (330 x *g*, 15 min, 20°C). The pellet was resuspended in Hepes-Tyrode buffer (0.145mM NaCl, 5mM KCl, 0.5mM Na2HPO4, 1mM MgSO4, 10mM Hepes, 5mM D-glucose, pH 6.5). To prevent aggregation prostacyclin (PGI2) was added (final concentration 10ng/ml) and platelets were washed again by centrifugation (330x *g*, 15 min, 20°C). The pellet was resuspended in Hepes-Tyrode buffer pH 7.2 to a final concentration of 2x10^11^platelets/L. The washed platelets were left at room temperature for thirty minutes to achieve a resting state before examining platelet function.

**Patients**

In patients treated with sunitinib plasma was obtained by centrifugation of a sodium citrate vacutainer (final conc. 3.2%) at 800x *g* for 15 minutes at 20°C, with subsequent centrifugation of the plasma at 2400x *g* for 10 min. Serum was obtained by centrifugation at least 30 minutes after collection of blood in a vacutainer without additive (1350x *g,* 15 minutes, 4°C). For isolation of PRP, platelet poor plasma (PPP) and platelets, blood was drawn in two 8.5ml ACD vacutainers containing 0.11 ml/L sodium citrate, acid citrate and glucose (Becton-Dickinson, Heidelberg, Germany; Cat. No. 364606). Platelet aggregation was analyzed in PRP, prepared by centrifugation of the vacutainer (156x *g,* 15 minutes, 20°C). A final concentration of approximately 2x10^11^platelets/L was obtained by diluting PRP with PPP (obtained by centrifugation of approximately one third of PRP (356x *g,* 15 min, 20°C)). Another ACD vacutainer was centrifuged at 160x *g* for 20 minutes at 20°C and PRP was then centrifuged at 356*x g* for 15 minutes at 20°C to obtain a platelet pellet and PPP. The platelet pellet, PPP, plasma and serum were stored in -80 degree Celsius.

From patients receiving a single administration bevacizumab blood was drawn in an ACD vacutainer. PRP (2x10^11^platelets/L) was processed as described above.

**Platelet aggregation**

Optical aggregation (standard test to examine platelet function) was monitored in a Chronolog lumiaggregometer (Chronolog, Haverford PA, USA) at 37°C at a stirring speed of 900 rpm. The influence of sunitinib, sorafenib and bevacizumab was studied *in vitro* on different platelet activation pathways by stimulating platelets by ADP, collagen, thrombin, arachidonic acid and ristocetin-activated vWF. Washed platelets were preincubated with sunitinib (10 or 20μM), sorafenib (5, 10, 25μM) or bevacizumab (50, 100 or 250μg/ml) for 10 minutes at 37°C. *Ex vivo,* the effect of sunitinib and bevacizumab was studied in PRP by stimulating platelets by ADP and collagen. One minute before activation platelets were stirred. Platelet aggregation was recorded for 15 minutes and the maximum value within this period was used. To be able to detect potential differences in aggregation due to antiangiogenic treatment, baseline aggregation levels of at least 30% were required, which meant for the *in vitro* experiments platelets without an antiangiogenic agent and for the *ex vivo* analyses platelets obtained before start of treatment. Besides, an aggregation level below 80% was used preferably. To achieve these levels, we initially activated platelets with different concentrations (ADP: 2.5, 5 and10μM; collagen: 0.25, 0.5, 0.75 and1.0μg/ml; thrombin: 0.125 and 0.25U/ml; arachidonic acid: 0.25mM; ristocetin/vWF: 25mg/ml/5μg/ml) and used the concentration of the agonist resulting in the highest aggregation level that was below 80% at all time points. *In vitro* aggregation experiments were performed in presence of fibrinogen (100μg/ml), except for those initiated by collagen and vWF/ristocetin.

**Flow cytometric analysis of platelet activation**

The impact of the antiangiogenic agents on platelet response to agonist stimulation was also determined by flow cytometry (BD FACSCanto II, Becton Dickinson), and measured as fibrinogen binding to integrin αIIbβ3 and surface expression of P-selectin on platelets. Briefly, washed platelets were incubated with sunitinib (20μM), sorafenib (25μM) or bevacizumab (250μg/ml) for 10 minutes at 37°C prior to stimulation with serial dilutions of ADP (0-250μM), CRP-xL (0-5000ng/mL) or PAR1-AP (0-1250μM) for 20 minutes in the presence of FITC-conjugated anti-fibrinogen, RPE-conjugated anti-CD62P and APC-conjugated anti-GP1b antibodies. Reactions were stopped by 1:100 dilution (v/v) in formyl saline (0.2% formaldehyde in 0.9%NaCl). Platelets were identified with forward and sideward scatter and Median Fluorescent Intensity (MFI) data were obtained.

**Tyrosine phosphorylation of c-Src**

To study if the TKIs sunitinib and sorafenib exert their actions on platelet function by affecting tyrosine phosphorylation, washed platelets were preincubated with sunitinib (20μM) and sorafenib (25μM) for 10 minutes at 37°C, stimulated with vehicle or collagen (1μg/ml) for 10 minutes at room temperature, and mixed (1:10 v/v) with ice-cold lysis buffer (10% (v/v) Nonidet P-40, 5 % (v/v) octylglucoside, 50 mM EDTA, 1% (w/v) SDS) supplemented with 5mM NaVO_3_ and 10% (v/v) protease inhibitor cocktail. c-Src was precipitated with protein G sepharose in combination with anti c-src (sc-8056; 1μg/ml), overnight at 4°C. Precipitates were washed 3 times with lysis buffer supplemented with 1mM phenylmethylsulfonyl fluoride (PMSF), 1mM NaVO3, and 1μg/mL leupeptin and samples were collected in reducing Laemmli sample buffer.

Tyrosine phosphorylation of c-Src was analyzed by SDS-PAGE and Western blotting. After blocking, the nitrocellulose membranes were incubated with the appropriate antibody (4G10 to detect tyrosine phosphorylation of c-Src; 1:2000, or anti src (sc-19) to detect src protein; 1:250) overnight at 4°C.

Antibody binding was detected using peroxidase-linked secondary antibodies (anti-rabbit alexa800 and anti-mouse alexa680, both 1:7500). For semiquantitative determination of phosphorylated or total protein, the bands were analyzed using ImageQuant software (Molecular Dynamics). Data express the semi-quantification of phosphorylation of c-Src relative to the density of the bands representing equal lane loading.

**Platelet-endothelial adhesion assay (real-time perfusion)**

Platelet-endothelial cell adhesion assays were performed as reported previously (1). In short, human umbilical vein endothelial cells (HUVECs, passage 2-3) were grown till confluence on glass coverslips in EBM2 Basal Medium supplemented with EGM-2 Single-Quots. Prior to perfusion coverslips were washed once in Medium 199. Washed platelets were incubated with 5 or 10μM sunitinib, with 10μM sorafenib or with 100 or 250μg/ml bevacizumab for 10 minutes at 37°C. A coverslip with HUVECs was also preincubated for one hour with 5μM sunitinib. 2x10^11^platelets/L were perfused over the HUVECs at a shear rate of 300 sec^-1^. Formation of vWF strings was visualized using an Axio Observer microscope (Zeiss). After 5 minutes of perfusion a snapshot was taken for quantification of the number of platelets adhered to a vWF string. The number was divided by the length of the string, resulting in the mean platelet coverage.

**Liquid chromatography-tandem mass spectrometry (LC-MS/MS)**

Analysis of sunitinib concentrations in serum and plasma was performed as reported previously (2). Briefly, chromatographic separation (Prodigy ODS-3; 3μm; 100mm×2.0mm) was conducted using tandem LC-MS/MS with a Turbo Spray Ionization source and an isocratic mobile phase. The mobile phase consisted of 66.6% acetonitrile:25% 20mM ammonium acetate (pH 7.8):8.3% methanol (v/v) at a flow rate of 200 µl/min with a 1µl injection volume and a 5 min run time. Data analysis was performed using Analyst version 1.42 from Applied Biosciences.

**Activation markers of endothelial cells and platelets**

Levels of beta-TG, P-selectin, RANTES, vWF and OPG were determined in citrate plasma samples in duplo using a semi-automatic ELISA in 384 wells format on a TECAN Freedom EVO (Tecan, Switzerland). For each antigen, Nunc maxisorp plates (Nunc, Denmark) were coated with the appropriate capture antibody and incubated overnight at 4°C (1µg/ml MAB393, 1µg/ml Duoset Dy137, 0.5µg/ml MAB278, 0.775µg/ml A0082, 1µg/ml MAB8051). Plates were blocked with 1% BSA/PBS and incubated. After a washing step, diluted citrate samples and calibration curves were added to the plate and incubated. The sample dilutions were made in 1% BSA/PBS: 1/80, 1/10, 1/10, 1/625 and 1/8, respectively. After a washing step, the detection antibody was added and incubated (0.05µg/ml BAF393, 0.01µg/ml Duoset Dy137, 1µg/ml AB-278-NA, 0.275µg/ml P0226, 0.1µg/ml BAF805). Depending on the type of detection antibody, HRP labelled streptavidin-HRP or a HRP labelled goat anti rabbit antibody was added after washing. For the vWF ELISA this last step was not necessary since HRP was directly labelled to the detection antibody. After a last washing step, luminol substrate was added and luminescence was measured in a Spectramax L luminometer (Molecular Devices) at 470 nm. All volumes used in the semi-automatic ELISA were 40µl, all incubations except with the capture antibody were done at RT while shaking. All washing steps were performed five times with 0.05%Tween/PBS.

**VEGF analysis**

VEGF concentrations were analyzed in serum, PPP and in lysed platelets (M-PER lysis buffer) with the R&D Systems Quantikine ELISA Kit according to the manual. Protein concentrations were analyzed using a BCA Kit.

**RESULTS**

**Patients**

Within six weeks after start, two patients discontinued sunitinib treatment. Reasons for discontinuation were rapid deterioration with confusion and weakness for one patient; and neurologic deficit, vomiting and diarrhea for the other patient. A temporary treatment interruption was necessary for seven patients in the first six weeks. Reasons for treatment interruption were: thrombocytopenia (grade two), viral infection, hand foot syndrome (HFS), hospital admission with malaise and fever, hospital admission with vomiting, presentation on the first aid with epistaxis together with malaise, nausea and dizziness, and for another patient gastrointestinal complaints together with epistaxis and malaise. These patients resumed treatment with a dose modification.

**References:**

1) Evelyn Groot, Rob Fynheer, Silvie AE Sebastian, Peter J Lenting, Philip G De Groot (2008) [Transition from Non-Platelet-Binding to Platelet-Binding Conformation of Von Willebrand Factor Occurs upon Exocytosis](http://www.bloodjournal.org/content/112/11/3917). Blood 112:3917

2) [Honeywell R](https://www.ncbi.nlm.nih.gov/pubmed/?term=Honeywell%20R%5BAuthor%5D&cauthor=true&cauthor_uid=20382575), [Yarzadah K](https://www.ncbi.nlm.nih.gov/pubmed/?term=Yarzadah%20K%5BAuthor%5D&cauthor=true&cauthor_uid=20382575), [Giovannetti E](https://www.ncbi.nlm.nih.gov/pubmed/?term=Giovannetti%20E%5BAuthor%5D&cauthor=true&cauthor_uid=20382575), [Losekoot N](https://www.ncbi.nlm.nih.gov/pubmed/?term=Losekoot%20N%5BAuthor%5D&cauthor=true&cauthor_uid=20382575), [Smit EF](https://www.ncbi.nlm.nih.gov/pubmed/?term=Smit%20EF%5BAuthor%5D&cauthor=true&cauthor_uid=20382575), [Walraven M](https://www.ncbi.nlm.nih.gov/pubmed/?term=Walraven%20M%5BAuthor%5D&cauthor=true&cauthor_uid=20382575), [Lind JS](https://www.ncbi.nlm.nih.gov/pubmed/?term=Lind%20JS%5BAuthor%5D&cauthor=true&cauthor_uid=20382575), [Tibaldi C](https://www.ncbi.nlm.nih.gov/pubmed/?term=Tibaldi%20C%5BAuthor%5D&cauthor=true&cauthor_uid=20382575), [Verheul HM](https://www.ncbi.nlm.nih.gov/pubmed/?term=Verheul%20HM%5BAuthor%5D&cauthor=true&cauthor_uid=20382575), [Peters GJ](https://www.ncbi.nlm.nih.gov/pubmed/?term=Peters%20GJ%5BAuthor%5D&cauthor=true&cauthor_uid=20382575) (2010) Simple and selective method for the determination of various tyrosine kinase inhibitors used in the clinical setting by liquid chromatography tandem mass spectrometry. [J Chromatogr B Analyt Technol Biomed Life Sci](https://www.ncbi.nlm.nih.gov/pubmed/20382575) 878:1059-1068. doi: 10.1016/j.jchromb.2010.03.010
