# Supplementary figures and images for: Platelet function is disturbed by the angiogenesis inhibitors sunitinib and sorafenib, but unaffected by bevacizumab
Source: Angiogenesis. 2018 Mar 12;21(2):325–34. doi: 10.1007/s10456-018-9598-5 (PMC5878190; doi:10.1007/s10456-018-9598-5)

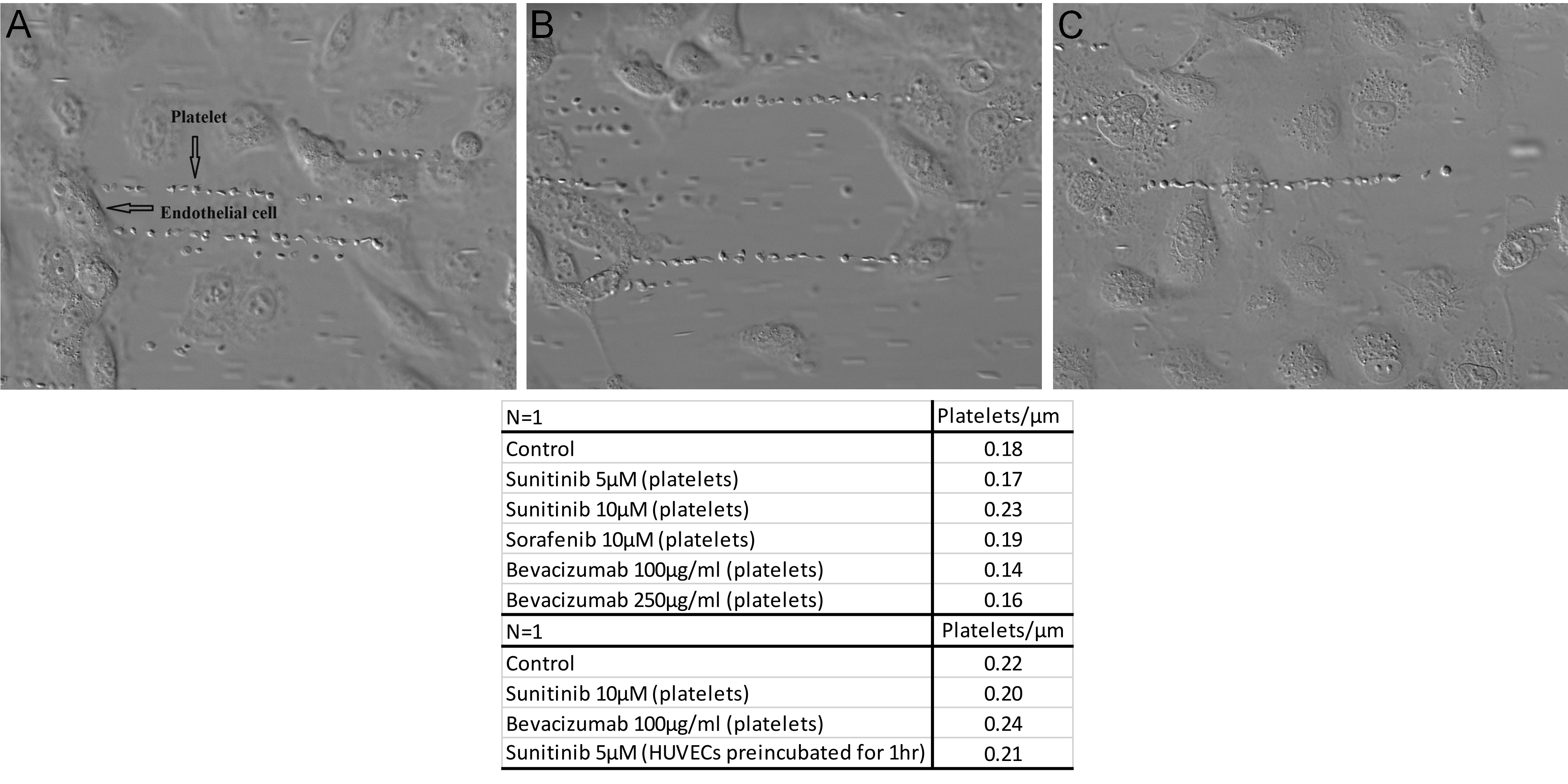

Supplement: Supplementary file 2 — Supplemental Figure 1: Platelet adherence to secreted vWF strings on stimulated endothelial cells under shear stress (by real-time perfusion). A) Platelets and HUVECs in absence of an angiogenesis inhibitor. B) Pre-incubation of platelets for 10 min with 10 μM sunitinib. C) Pre-incubation of HUVECs for 1 h with 5 μM sunitinib. The number of platelets attached per micrometer vWF string is presented in the quantification (JPEG 2621 kb) [file 10456_2018_9598_MOESM2_ESM.jpg]

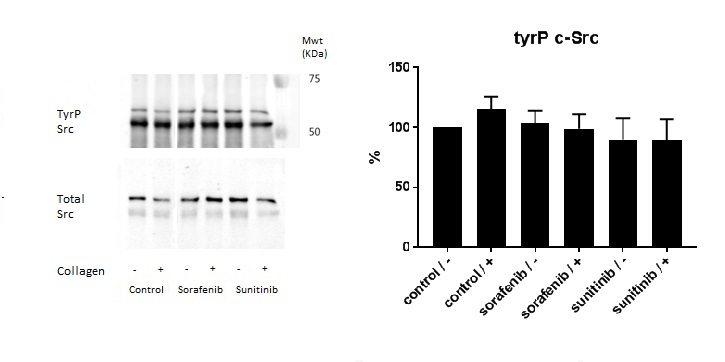

Supplement: Supplementary file 3 — Supplemental Figure 2: Tyrosine phosphorylation of c-Src in the presence of sunitinib and sorafenib. Platelets were stimulated with collagen after treatment with vehicle, sunitinib or sorafenib. Tyrosine phosphorylation of c-Src was determined by SDS-PAGE after immunoprecipitation from platelet lysates (left, upper panel). An antibody against c-Src was used as a control for equal lane loading (left, lower panel). Src has a molecular weight of approximately 60KDa. The graph (right panel) shows the semiquantification of tyrosine phosphorylation of c-Src. Data are expressed as percentage of total c-Src protein. (N = 3) (JPEG 34 kb) [file 10456_2018_9598_MOESM3_ESM.jpg]
