# Supplementary material for: Platelet function is disturbed by the angiogenesis inhibitors sunitinib and sorafenib, but unaffected by bevacizumab
Source: Angiogenesis. 2018 Mar 12;21(2):325–34. doi: 10.1007/s10456-018-9598-5 (PMC5878190; doi:10.1007/s10456-018-9598-5)
Supplement: Supplementary file 4 — Supplemental Table 1A: Details of unavailability of platelet aggregation data for the agonists ADP and collagen from patients treated with sunitinib (A). 1 = Distinct thrombocytopenia; 2 = Interruption or discontinuation due to toxicity/progressive disease; 3 = Technical problems; 4 = No blood was drawn; 5 = Pretreatment aggregation level below 30%. Use of co-medication that might influence hemostasis are included (PDF 120 kb) [file 10456_2018_9598_MOESM4_ESM.pdf]

	Collagen			ADP			Co-medication
Patients	24hr	3wk	6wk	24hr	3wk	6wk	
1	No (3)	No (1)	No (2)	Yes	No (1)	No (2)	Acetylsalicylic acid
2	Yes	Yes	Yes	Yes	Yes	No (3)	
3	Yes	Yes	Yes	Yes	Yes	Yes	
4	Yes	Yes	Yes	Yes	Yes	Yes	
5	No (3)	No (2)	No (2)	No (3)	No (2)	No (2)	
6	Yes	Yes	No (4)	Yes	Yes	No (4)	
7	Yes	No (2)	No (2)	Yes	No (2)	No (2)	
8	Yes	Yes	Yes	Yes	Yes	Yes	Fraxodi
9	Yes	No (3)	No (3)	No (5)	No (5)	No (5)	
10	No (5)	No (5)	No (5)	No (3)	No (2)	No (2)	
11	No (5)	No (5)	No (5)	No (5)	No (5)	No (5)	Acetylsalicylic acid
12	No (5)	No (5)	No (5)	Yes	Yes	Yes	Acetylsalicylic acid
13	No (5)	No (5)	No (5)	No (5)	No (5)	No (5)	Fraxodi
14	No (5)	No (5)	No (5)	Yes	No (2)	No (2)	Acetylsalicylic acid
15	No (5)	No (5)	No (5)	Yes	Yes	Yes	Fragmin
16	No (1/5)	No (1/5)	No (1/5)	No (1/5)	No (1/5)	No (1/5)	
17	Yes	No (2)	No (2)	Yes	No (2)	No (2)	
