# Supplementary material for: Platelet function is disturbed by the angiogenesis inhibitors sunitinib and sorafenib, but unaffected by bevacizumab
Source: Angiogenesis. 2018 Mar 12;21(2):325–34. doi: 10.1007/s10456-018-9598-5 (PMC5878190; doi:10.1007/s10456-018-9598-5)
Supplement: Supplementary file 5 — Supplemental Table 1B: Details of unavailability of platelet aggregation data for the agonists ADP and collagen from patients treated with bevacizumab (B). 1 = Distinct thrombocytopenia; 2 = Interruption or discontinuation due to toxicity/progressive disease; 3 = Technical problems; 4 = No blood was drawn; 5 = Pretreatment aggregation level below 30%. Use of co-medication that might influence hemostasis are included (PDF 119 kb) [file 10456_2018_9598_MOESM5_ESM.pdf]

	Collagen		ADP		Co-medication
Patients	5hrs	day 3-5	5hrs	day 3-5	
1	No (3)	Yes	No (3)	Yes	
2	Yes	Yes	No (5)	No (5)	
3	Yes	Yes	Yes	Yes	
4	No (1)	Yes	No (1)	Yes	Acenocoumarol
5	No (5)	No (5)	Yes	Yes	
6	Yes	Yes	Yes	Yes	
7	Yes	Yes	Yes	Yes	
8	No (5)	No (5)	Yes	Yes	Acetylsalicylic acid
