# Supplementary material for: Platelet function is disturbed by the angiogenesis inhibitors sunitinib and sorafenib, but unaffected by bevacizumab
Source: Angiogenesis. 2018 Mar 12;21(2):325–34. doi: 10.1007/s10456-018-9598-5 (PMC5878190; doi:10.1007/s10456-018-9598-5)
Supplement: Supplementary file 6 — Supplemental Table 2: Concentrations of sunitinib in plasma and in serum, measured by LC–MS/MS at 24 h and 3 weeks after start of treatment. N is the number of patients, SEM is the standard error of mean (PDF 142 kb) [file 10456_2018_9598_MOESM6_ESM.pdf]

	Concentration Sunitinib (nM)	
	24hr	3wk
Plasma	78.3	151.8
N	17	9
SEM	9.7	21.3
Serum	99.2	191
N	17	8
SEM	13.2	30.2
